# How to Facilitate Decision-Making for Hematopoietic Stem Cell Transplantation in Patients With Hemoglobinopathies. The Perspectives of Healthcare Professionals

**DOI:** 10.3389/fped.2021.690309

**Published:** 2021-08-18

**Authors:** Hilda Mekelenkamp, Herma van Zanten, Martine de Vries, Arjan Lankester, Frans Smiers

**Affiliations:** ^1^Department of Pediatrics, Willem-Alexander Children's Hospital, Leiden University Medical Centre, Leiden, Netherlands; ^2^Department of Medical Ethics and Health Law, Leiden University Medical Centre, Leiden, Netherlands; ^3^Department of Obstetrics, Leiden University Medical Center, Leiden, Netherlands

**Keywords:** decision-making, hematopoietic stem cell transplantation, health care professional, hemoglobinopathy, sickle cell disease, Thalassemia

## Abstract

Hematopoietic stem cell transplantation decision-making for hemoglobinopathy patients is a complex process, and it remains difficult for health care professionals to decide whether and when a hematopoietic stem cell transplantation should be offered. Gaining insight into health care professionals' considerations is required to understand and optimize this decision-making process. A qualitative interview study using semi-structured interviews with eighteen health care professionals. Data were thematically analyzed. Two main themes emerged from the data: (1) *Experiencing the influence of a frame of reference* and (2) *Feeling responsible for a guided decision-making*. The frame of reference, meaning the health care professionals' knowledge and experiences regarding hematopoietic stem cell transplantation, influenced the guided decision-making process. Subsequently, three subthemes evolved from the second theme: (a) weighing up disease severity against possible complications, (b) making an effort to inform, and (c) supporting the best fitting decision for the individual patient. The health care professionals' frame of reference determined the hematopoietic stem cell transplantation decision-making process. This demands reflection on the health care professionals' own frame of reference and its influence on decision-making. Furthermore, reflection on the frame of reference is needed by exchange of knowledge and experiences between referring and referred-to healthcare professionals in an open and two-way direction. The transplantation teams have a responsibility of keeping the frame of reference of their referring colleagues up to date and referring health care professionals should share their feelings regarding hematopoietic stem cell transplantation. To guide patients, a shared decision-making approach is supportive, in which eliciting the patients' preferences is highly important. Health care professionals can refine the decision-making process by guiding patients in eliciting their preferences and including these in the decision.

## Introduction

Hemoglobinopathies are one of the most common recessive diseases affecting humans worldwide. At least 5.2% of the global population is a carrier of hemoglobinopathy (1). Annually, over 330.000 affected children are born, 17% have thalassemia, and 83% have Sickle Cell Disease (SCD) (1). Despite improvements in supportive care for both diseases, life expectancy and quality of life remain severely hampered (2–4). Allogeneic hematopoietic stem cell transplantation (HSCT) offers an established curative option (5–9) but is associated with significant risks (6, 10). Gene therapy is a promising alternative treatment modality, but long-term outcome data are currently lacking (11). The process to decide for HSCT, gene therapy, or remaining on supportive care is complex (12, 13).

In current practice, it remains difficult for healthcare professionals (HCPs) to decide when HSCT should be offered as an option in hemoglobinopathy patients (10, 14–16). As there is no clear-cut general answer to what the best treatment would be, the patients' and/or families opinions are of high value to include in decision-making. In general two types of medical decisions can be distinguished, *effective*, and *preference-sensitive* decisions (17). Effective decisions refer to decisions in which a scientific certainty exists, and clearly more pros than cons are known. Preference-sensitive decisions point to decisions in which no clear-cut answers are available, and the pros and cons are dependent on individual values (17). Treatment decision-making in hemoglobinopathy patients can be approached as preference-sensitive (18). A shared decision-making (SDM) approach can facilitate preference sensitive decisions (19, 20). This approach based on autonomy principles, aims to involve the patient's preferences and values in a collaborative decision-making process. An SDM approach in pediatrics showed to decrease uncertainty, clarify the patient's future health status, and facilitate the making of high-quality decisions (21–23).

For SDM to start, at least one of the participants (patient, family, HCP) needs to bring up the subject. Currently, it is unclear how HCPs decide whether HSCT for a given patient should be offered or explored (24). From clinical practice, fear of the unknown or personal opinions seem important issues influencing the position of HCPs in the decision-making process. Gaining insight into HCPs' considerations and whether these promote or hamper SDM is important because HCPs are involved in starting the process of deciding. This study aims to identify the considerations used by HCPs in the HSCT decision-making process in patients with hemoglobinopathies and/or caregivers.

## Methods

### Study Design

A qualitative interview study to identify and describe HCP's perspectives was conducted. As this study aimed to make sense of the perspectives of HCPs and very little is known about these perspectives, a qualitative method was chosen. A qualitative approach provides the opportunity to be able to describe, understand, and interpret the perspectives of HCP's (25, 26).

### Population and Recruitment

This study is part of a longitudinal, multi-center study, focusing on the HSCT decision-making process from the patients' perspectives (0–35 yr) with hemoglobinopathies, their caregivers, and the involved HCPs. The six largest expert centers on both (pediatric and adult) regular hemoglobinopathy care and HSCT in the Netherlands participated in this study. These six specialized centers collaborate in SCD research in the Netherlands as the so-called SCORE consortium. The results of the interviews with the involved HCPs are described in the present article. Using purposive sampling, hematologists, transplantation specialists, and nurse practitioners from the six involved expert centers and actively involved in the care of the included patients in the larger study were invited by mail to participate. Purposive sampling was chosen in order to select professionals with extensive experience in hemoglobinopathies and HSCT. We intended to achieve a maximum variation in years of experience in the field and experience as referring hematologist, nurse specialist, and HSCT specialist. In consistence with qualitative research standards, inclusion continued until thematic saturation was reached (26). Saturation was reached after 17 interviews since the analysis of the last interview (interview 18) did not add to the descriptions of the themes and no new themes emerged (27). This number of respondents is in agreement with qualitative research standards, where 14–20 subjects are seen sufficient in heterogeneous groups (26). All study procedures were in accordance with the declaration of Helsinki (28), and all subjects consented to participation in the study. The medical ethics committee of Leiden University Medical Center approved the study protocol (P17.084).

### Data Collection

A nurse specialized in pediatric HSCT with experience in qualitative interviewing (HM) conducted the individual semi-structured interviews. Semi-structured interviews provide the opportunity to gain insight into the HCPs' considerations, whereas at the same time the focus of the interview can be controlled. A topic list was used to ensure whether all topics to answer the research question were covered (Table 1) (26, 27). This topic list was based on preliminary studies on HSCT decision-making and experts' experiences (23, 29–32). The topic list was evaluated and adjusted throughout the project twice, based on insights from the interviews and analyses, referring to an iterative process (26). The interviewed HCPs were involved in the decision-making process of specific patients (included in the larger study), either as (referring) pediatric hematologist, HSCT physician or nurse practitioner. The starting point for the interviews was this specific patient case. For examples of such patient cases, see Supplementary Table 1. Interviews consist of open-ended questions. The interviews were audio-recorded and transcribed verbatim. Observational memos were used to describe the setting, atmosphere, circumstances, and the researchers' reflections on the interview themes (26).

**Table 1:** Topic list.

**Topic list interviews**
**Openings question** Could you tell me about the history and involvement with this patient/family? What did the treatment trajectory look like?
**Conditioning and treatment trajectory of a specific patient** •Could you tell me something about the illness course of this patient? •How would you describe your contact with the patient or family? •What goals would you like to achieve with the patient or family? •To what extent does the patient or family have insight into treatment, complications, and treatment options? •Could you describe your approach to increase insight into the illness, complications, and treatment possibilities? Do you use a specific methodology? •What are your thoughts about the current treatment options for [sickle cell disease or thalassemia]? What causes you to think about these treatment options in this way? •What are your expectations regarding this patient? •How are (treatment) decisions made for this patient, family?
**The process of treatment decision-making** •How did the conversation with your patient come to the stem cell transplantation come? •How did the process of decision-making toward a possible stem cell transplantation work out for this family?^*^ •*Who had a role in the decision for a possible stem cell transplantation for this patient?* •*What was the extent of this role (in percentages) for each individual and how do you feel about the division of roles?* •*What do you personally think will be the best choice of treatment for this child/patient?* •*Is it important to you that the child will be able to make their own choices?* •Do you use a guideline to determine which patient or families you will inform about a possible stem cell transplantation and, if so when? •Could you describe whether (or not) you use a set counseling structure when informing/counseling about stem cell transplantation? •How do you generally talk with patients about their treatment decisions? •In what way do you counsel patients in a situation where there is no one-size-fits-all answer as to the best treatment option?
**HCPs' considerations when discussing HSCT with a patient** •Which factors do you consider when talking to a patient about stem cell transplantation? •Could you describe if there are situations in which you will refrain from informing the patient/family about stem cell transplantation? What are the most important considerations in that case? •Could you describe how you involve both child and parents?
**Influencing factors in HSCT decision-making** •What is your view on a stem cell transplantation for patients with sickle cell disease or thalassemia? •What are your experiences of stem cell transplantation? •*How would you describe the decision for a stem cell transplantation for this child (explanation of preference-sensitive and effective decisions)? And for sickle cell disease and thalassemia in general?* •*In case of a preferred decision, how do you involve the patient in the decision? Is this different in the case of an effective decision?* •*Does the type of donor also play a role in this?* •*Does the type of conditioning play a role for you (myeloablative vs. non-myeloablative)?* •*How do you view gene therapy?* •*Patients indicate that they would like to talk to other patients with experience with a stem cell transplantation. What do you think about this and do you have any ideas on how to do this?*
**Guidance of patients and/or caregivers** •Could you describe what information options you use? •What methods of guidance and counseling do you consider to be most effective? •Do you have any suggestions regarding information/counseling?

* *Questions in italic were added during the research process*.

### Data Analysis

Thematic analysis was used to identify patterns and themes that reflected the HCPs' perspectives (25–27, 33). Transcripts were read and reread for familiarization with the data and the generation of initial codes (HM,HZ). Open coding was conducted independently by two team members (HM,HZ). During this stage the transcripts were segmented into meaningful parts and labeled (coded) with a description of the essence of that part of the text. Subsequently, focused coding was used, comparing codes and categorizing these on a more conceptual level. Finally, categories were described into themes, interpreting the data and describing the meaning of the categories (33, 34). The themes were described in the results section and represent the considerations of the total group of HCPs. When differences did appear within the subgroups of HCPs, this is indicated. Discrepancies in coding, the developing of categories, and defining and refining of the themes were discussed until consensus was reached (HM,HZ,MV). The research team was involved in all phases of data collection and analysis to enhance validity and credibility (27). The COREQ checklist was used to enhance comprehensive reporting (Supplementary Table 2) (35). Coding was supported by qualitative data analysis software, ATLAS.ti (version 8) (36).

## Results

Between July 2017 and January 2020, 18 HCPs, including 10 (pediatric) referring hematologists, five transplantation specialists from HSCT centers, and three nurse specialists from referring centers, were approached and participated in the interviews (Table 2). The mean age of the professionals was 47 years (SD 9; range 33–64 years); the professional experience of the HCPs in the field of hemoglobinopathy was variable with a mean of 10 years (SD 8; range 1–29 years). Each HCP was interviewed once, and most interviews took place at the HCP's office and one by phone. The interviews lasted on average 38 min (SD 9; range 27–57 min). Two main themes emerged from the qualitative analysis of the interviews: (*1*) *experiencing the influence of a frame of reference* and (*2*) *feeling responsible for a guided decision-making process*. The first theme appeared to be an overarching theme, and the way how this frame of reference influenced HCPs in their decision-making is described within the second theme. This second theme is split in three subthemes. For illustrative quotations (37), see Table 3.

**Table 2:** Characteristics.

**Respondent**	**Center**	**SCD/TDT population in center**	**Experience in the field of hemoglobinopathy**	**Specialized in SCD/TDT**	**Specialized in HSCT incl. for hemoglobinopathy patients**	**Nurse specialist**
HCP 1	1	>200	18	Yes	No	No
HCP 2	3	<50	14	Yes	Yes	No
HCP 3	3	<50	7	Yes	Yes	No
HCP 4	2	100–200	10	Yes	No	No
HCP 5	2	100–200	5	Yes	No	Yes
HCP 6	1	>200	5	Yes	No	No
HCP 7	1	>200	3	Yes	Yes	No
HCP 8	1	>200	15	Yes	No	Yes
HCP 9	1	>200	4	Yes	No	No
HCP 10	6	<50	17	Yes	Yes	No
HCP 11	6	<50	1	Yes	Yes	No
HCP 12	6	<50	4	No	Yes	No
HCP 13	6	<50	2	No	Yes	No
HCP 14	1	>200	29	Yes	No	No
HCP 15	1	>200	3	Yes	No	Yes
HCP 16	5	50–100	2	Yes	No	No
HCP 17	5	50–100	22	Yes	Yes	No
HCP 18	4	100–200	15	Yes	No	No

**Table 3:** Illustrative quotations.

**Quotations illustrating the relationship between a frame of reference (theme 1) and a guided decision-making process (theme 2)**		
**Subtheme**	**Situation**	Quotation^*^
2A Weighing up disease severity against possible complications	A referring hematologist with experience as a HSCT specialist, talking about transplantation criteria for hemoglobinopathy patients. (HCP3)	“*I think, but that is presumably true for all transplantation specialists, we are all very much inclined to do perform a transplant sooner because we see how safe transplanting is […]. I think the whole vasculopathy part, that makes me think the sooner the better, that is not something you would want to do. And of course, there are the risks of HSCT, there is the long-term vascular damage, so I think that patients should have as minimal visible symptoms as possible, those still existing.”*
	A referring hematologist talking about the need for knowledge sharing by HSCT centers regarding results in hemoglobinopathy patients and remembering previous patients. (HCP18)	“*I have already seen several patients in the past with quite serious HSCT complications such as the need for liver transplantation and more of such problems. I don't know if I would want to expose my patients to these risks. I struggle with that.”*
	A referring hematologist talking about how he is dealing with the opinions of his team about HSCT complications. (HCP16)	“*Infertility is always brought up as a HSCT complication, by my colleagues and that is a serious point. Sometimes, I wonder about the importance of infertility. It is important, of course. But there is a disease that needs to be cured. But that's my opinion, and then of course there are also possibilities for cryopreservation.”*
	An HSCT specialist' vision on HSCT for hemoglobinopathy patients. (HCP13)	“*I can support (that) given that I'm obviously trained in an HSCT center and very much taken their vision. But if there is a suitable donor with a healthy child, a good starting point then I would support the idea of early transplantation for it could significantly improve the long term quality of life, because one could limit and prevent the iron overload and such kind of misery. You could actually cure a child of his illness and its chronicity, to then sincerely hope that it may be an uncomplicated trajectory. And a complicated trajectory is especially very difficult in this category, but you will not know this ahead of time.”*
2B Making the effort to inform	Referring hematologist talking about the importance of informing families and to be as neutral as possible. (HCP1)	“*I would never say, as a doctor, you would ask me, what would you advise me. Yes, it's not important what I think, but it's important what you think, and your child would think and the reasons for which you decide whether you want something or not. This idea needs to develop and if, for example, you need to go an HSCT center to know exactly what it would involve.”*
	Referring hematologist about the difference for HSCT indication for transfusion dependent thalassemia and sickle cell disease. (HCP14)	“*Yes, I have too many bad experiences. I experienced that children died or ventured from one chronic disease into the other chronic disease, while their quality of life really was reduced. So, I do not have a positive experience with children having sickle cell disease. So, I'm pretty conservative about that.”*
	An HSCT physician speaking about the HSCT decision-making process. (HCP13)	”*I would prefer them to say We are the ones deciding because we are very well-informed, and we know what to decide about. That would be the best possible answer. Yes. And we would have, largely, done a good job.”*
	Referring hematologist speaking about her own thoughts on HSCT for a specific patient who asked for more information about an HSCT. (HCP9)	“*Because if I would think for myself, well X, with auto immune hemolytic anemia in your medical history, tough. On the other hand, he meets the HSCT eligibility criteria. So, I have been considering, why have I not mentioned this sooner? Well, I don't know, there is so much to discuss in a consultation.”*
2C Supporting for the best fitting decision for the individual patient	Referring hematologist supporting families to make the best decision for themselves in order to prevent for anticipated regret. (HCP 6)	“*I try to encourage people to seriously get the information, because I think it's really important for them if they have considered an HSCT, that they can really decide to go ahead with it or not with all the information. This would allow them to say, 10 years ago, we considered it for you, but, at the time, the information was leading us to make the decision not to offer it to you. Allow them that they could really justify that for themselves.”*
	Referring hematologist with experience as an HSCT specialist talking about medical care goals for a specific patient. (HCP3)	“*My goal for them is to provide good medical care and I believe this meaning an HSCT for patient X, which will give her the best chance.”*
	An HSCT specialist speaking about giving advice during counseling for HSCT. (HCP12)	“*It really has to be shared decision making, the risk that you as a doctor throw all the information at them and would say go ahead and find it out yourself, while that's not good either in my opinion. So, in that case you have to guide those people in the decision you have to make.”*
	Referring hematologist talking about the need for psychosocial support alongside the HSCT trajectory. (HCP 14)	“*I think at the moment you set the indication for transplantation you also need to look at the social problems or the psychosocial problems. So, I think as soon as you start the trajectory, you should immediately start the psychosocial trajectory.”*

* *The quotations are somewhat edited for legibility and anonymity*.

### Theme 1: Experiencing the Influence of a Frame of Reference

From the interviews, it became clear that their frames of reference influenced HCPs' considerations about HSCT decision-making for hemoglobinopathy patients. The frame of reference refers to *knowledge* and *experiences* of the HCPs, as mentioned in the interviews. Firstly, HCPs referred to *knowledge* on treatment and specifically curative treatment options. This knowledge can be the knowledge available to themselves, within their team, in the literature or from new emerging therapies. Furthermore, HCP's pointed to the lack of evidence about curative treatments such as RCTs. The HCPs' knowledge depends on their specialty and experience and therefore differs between referring specialists and HSCT specialists, but also juniors and seniors. Available knowledge on HSCT seems more difficult to translate into practice by referring HCPs without experience in HSCT, pointing to the influence of *experiences* on the HCPs' frame of reference. HCPs referred to either positive or negative (team) experiences with the disease or with HSCT. Most referring pediatric HCPs struggled with experiences of individual HSCT patients without having the overview on cohort level, as articulated by a referring HCP “*I have already seen several patients in the past with quite serious HSCT complications such as the need for liver transplantation and more of such problems. I don't know if I would want to expose my patients to these risks*.” At the same time, HCPs of adult patients struggled with the experiences of non-transplanted SCD patients with serious organ damage and impaired QoL. Experiences furthermore are different among junior or senior members. Junior team members explained that if solid evidence is lacking and they do not have extensive experience themselves, they rely even more on a shared decision-making approach in which they valued the voice of the patient or family crucial. Although, senior professionals also stated the importance of including the patients perspective into the decision, they rely on their experiences in feeling comfortable to offer (HSCT physicians) or being reluctant in discussing a HSCT (referring HCP). Besides, (junior) HCPs experienced the influence of opinions of other (senior) team members in the difficulty of weighing chances vs. risks when considering to refer a patient for HSCT. This influence was illustrated by examples HCPs gave during the interviews about the negative experiences their colleagues had with specific patients with severe HSCT complications. HSCT for transfusion-dependent thalassemia (TDT) patients appeared to be a more broadly accepted approach based on experienced good outcomes in previously transplanted TDT patients, more available evidence in the literature, and a more predictable disease course with non-curative therapy.

Despite their knowledge and experiences, most HCPs stated that guiding the patients objectively in their decision-making is of high importance. Nevertheless, the HCPs' frame of reference influenced their considerations and consequently their decision-making. The influence of the HCPs' frame of reference on decision-making is demonstrated in how HCPs guide their patients during the decision-making process.

### Theme 2: Feeling Responsible for a Guided Decision-Making Process

HCPs referred to treatment decision-making for patients with hemoglobinopathies as a process, starting with the first consultation where diagnosis, treatment, and curation possibilities are explained. During this phase, HCPs guide patients in coping with the disease, aiming for acceptance of the disease, and prevention and management of symptoms. In case of TDT, the possibility of HSCT is discussed with caregivers relatively early, at a young age of the child. For SCD, the option of an HSCT will usually be brought up in case of increased disease severity, for example, after an acute chest syndrome or in case of high risk of stroke. Increasingly, HCPs experience that pediatric and adult SCD patients or their caregivers themselves initiate the discussion on HSCT as a curative treatment option, often influenced by social media. Within the guided decision-making process, three subthemes emerged that describe the responsibility experienced by the HCPs in guiding patients and how their frame of reference promoted or hampered decision-making.

### 2A Weighing up Disease Severity Against Possible Complications

HCPs mentioned that, in general, they based the indication for HSCT on (inter)national criteria, and the HSCT teams made a final decision on the indication. In case caregivers have major doubts about an HSCT for their child with TDT and choose to continue with the transfusion scheme, most HCPs created time for parents to consider alternative options, or the HCPs accepted the caregiver's viewpoint. Most HCPs explained that transplanting TDT patients at a young age is important to prevent transfusions complications. Due to developments in transfusion and chelation therapy some HCPs reported a shift in their thinking, where the possibility of an HSCT should be explored, and the alternatives. When considering an HSCT for SCD patients, referring HCPs struggled with the dilemma between disease severity, the unpredictability of the disease process, the possible good responses on supportive care, and the significant risks of long-term HSCT complications. HCPs realized however, that non-curative treatments do not resolve the long-term disease burden sequelae, impacting the quality of life and life expectancy. Generally, all HCPs characterized the decision for HSCT as complex, weighing chances for cure vs. risks. HSCT physicians tend to offer HSCT more easily to patients meeting consensus-based criteria, whereas referring HCPs are more reluctant, both based on their formed frame of reference.

### 2B Making the Effort to Inform

To facilitate patients in decision-making, all HCPs emphasized their key role in providing the patient with objective information about all treatment options rather than giving advice. Most referring HCPs stated that they provided and discussed information about treatment possibilities regularly during the treatment over the years. HCPs explained that questions of patients or caregivers about curative therapy need to be respected and supported by providing all information. Therefore, patients were easily referred to an HSCT specialist to inform them with up-to-date knowledge about the currently used treatments and its outcomes. Nurse practitioners said they did not have a significant role in informing about HSCT, but patients regularly discussed their thoughts and questions about treatment possibilities with them. Their role appeared to be more supportive in decision-making rather than informative and decisive. In their counseling for a possible HSCT, HCPs included several aspects: the availability of a suitable donor, timing for a possible HSCT, future treatment possibilities and the perceived disease burden without HSCT, however hard to estimate and quantify. These latter aspects could have more or less impact depending on the disease severity, the families' wishes and circumstance, and were influenced by the HCPs' own frame of reference. As illustrated by a referring HCP: “*Because if I would think for myself, well X, with autoimmune hemolytic anemia in your medical history, tough. On the other hand, he meets the HSCT eligibility criteria. So, I have been considering why have I not mentioned this sooner? Well, I don't know, there is so much to discuss in a consultation.”* Although most HCPs stated that they have the task to inform, they based the timing of informing and discussing the option of HSCT on their frame of reference.

### 2C Supporting the Best-Fitting Decision for the Individual Patient

HCPs underlined their task in the SDM process in providing detailed information on an individual level. All HCPs stated that their role in the decision-making process is important, but most of them believed that neutrality is necessary to enable families to make their own decisions and to prevent for decisional regret. Since giving advice appeared to be influenced by personal experiences, some HCPs felt not experienced enough to fulfill this task. In contrast, other HCPs explained that they give some advice, mainly based on their experiences with HSCT, but also directed by the disease burden. In supporting patients in their decision-making, a trustful relationship with patients was mentioned as a vital condition; for patients to discuss their fears and for HCPs to understand patients' needs during the whole process. Furthermore, HCPs expressed the importance of paying attention to the patient's medical and psycho-social situation, treatment compliance, and the perceived burden of the disease. Providing support in such circumstances was considered essential by HCPs before proceeding in the HSCT decision-making process. This support seems essential for, but not limited to, patients with complex circumstances, such as a refugee background, adopted children or families with lower health literacy. Especially in adult patients' compliance was mentioned as of great importance for an intense treatment like HSCT, to increase the likelihood of a successful HSCT. HCPs mentioned that they consider the child's own wishes by not just speaking with the family but also, depending on his/her age, listening to the child's ideas and questions. The importance of team collaboration between the referring hematologists and the HSCT teams was mentioned in order to reach a consensus about the patient's disease progression, indication for HSCT, and proper support for the patient and/or family.

## Discussion

Our study identified “the influence of a frame of reference” as the central theme in the HCPs' decision-making process when considering an HSCT for hemoglobinopathy patients. This frame of reference referred to the HCPs' knowledge and experiences and influenced the way HCPs guided their patients in the HSCT decision-making process and therefore demands awareness.

This study showed that HCPs considered SDM as an important approach in HSCT decision-making for hemoglobinopathy patients. We described that HSCT for TDT patients, based on successful experiences and available knowledge on outcomes, was considered an effective treatment and therefore, as a less complex decision. In contrast, the choice for HSCT in SCD patients was mainly experienced as a preference-sensitive decision, and hence the decision-making process was more multifaceted. The HCPs were aware that advances in HSCT care indications for transplantation for both diseases are changing, complicating the decision-making process, and urging the need for SDM. Furthermore, disease burden, expected disease course, and possible HSCT-related complications were important factors in weighing a possible HSCT for patients. The more broadly accepted approach for HSCT in TDT patients compared to SCD patients confirmed the results of a previous study investigating HCPs' referral policy for HSCT in hemoglobinopathy patients (32). We observed that HCPs used a more advising-directed approach in case of TDT or severe complications in SCD, which was observed in a previous study. This study reported a collaborative or proponent approach in the process of decision-making for SCD patients, influenced by disease severity, the intensity of treatment, and urgency of treatment (38). Our study added to this mechanism with the strong influence of the HCP's frame of reference in guiding the decision-making process. The influence of training was observed previously, showing that the period of specialty training influenced the referral of patients for HSCT evaluation (32). Our study provided in-depth insight into how experience and knowledge influenced the HSCT decision-making process.

An important finding in our study is the influence of the HCPs' frame of reference. This finding leads to a pertinent message for clinical practice. Although some HCPs expressed they want to use a neutral approach, the question arises if and to what extent it is possible to maintain a neutral position (39). In order to reflect on neutrality, some aspects can be taken into consideration. First of all, to be aware of your own frame of reference, to prevent framing of available treatment options (39). Subsequently, the frame of reference demands a reflection from HCPs on this phenomenon by exchanging knowledge and experiences of referring and referred-to HCPs in a two-way direction. The HSCT teams have a responsibility of keeping the frame of reference of their referring colleagues up-to-date, by providing them outcomes and experiences on the cohort level. Referring HCPs should share their feelings regarding HSCT and discuss their patients with HSCT centers regularly, in an open peer-review way. The HSCT decision-making process will benefit from such an open collaboration between centers. Furthermore, the non-neutral position of the information giver can be overcome by involving multiple information givers in the decision-making process with their own approach and vision. Finally, an SDM approach is a supportive and guiding methodology. SDM goes beyond providing information on which patients can base their decision. Deliberating options with patients, eliciting their preferences, and including these in the decision are of equal importance (19, 20). Research evaluating the SDM approach in conversations showed low or moderate levels of patient-involving behavior. (40, 41) These results shows the need for the implementation of SDM interventions and SDM training for HCP. In treatment decision-making, an advocating role of the HCP is necessary in which both the patient's health and respect for the patient's self-determination need to be thoughtfully balanced (39).

A strength of our study was the ability to provide a broad view of perspectives from HCPs from multiple centers and with different backgrounds. Maximum variation in the sample was achieved, increasing the likelihood of representing the population. We enhanced the objectivity of the data analysis by double, independent coding and the involvement of experts on the topic in the research team. We did not perform data validation by member check, although discussion of the study results with the study population (SCORE consortium) confirmed our observation of the importance of the frame of reference. We had a relatively long inclusion period combined with new developing therapies, such as gene therapy. With our description of the influence of the frame of reference, we showed that knowledge is an important factor in considering treatment options for hemoglobinopathy patients. Knowledge regarding new therapies is rapidly evolving, showing that knowledge in SDM constantly improves and easily changes.

In conclusion, our study shows the influence of the HCPs' frame of reference on how they guide their hemoglobinopathy patients in the HSCT decision-making process. This decision-making process will benefit from the HCPs' reflection on their frame of reference and exchange of knowledge and experiences. Finally, HCPs can refine the decision-making process by guiding patients in eliciting their preferences and including these in the decision.

## Data Availability Statement

The raw data supporting the conclusions of this article will be made available by the authors, without undue reservation.

## Ethics Statement

The studies involving human participants were reviewed and approved by METC Leiden-Den Haag-Delft. Written informed consent for participation was not required for this study in accordance with the national legislation and the institutional requirements.

## Author Contributions

HM, MdV, AL, and FS contributed to conception and design of the study. HM performed the interviews. HM and HvZ analyzed the data and wrote the first draft of the manuscript. All authors contributed to manuscript revision, read, and approved the submitted version.

## Conflict of Interest

The authors declare that the research was conducted in the absence of any commercial or financial relationships that could be construed as a potential conflict of interest.

## Publisher's Note

All claims expressed in this article are solely those of the authors and do not necessarily represent those of their affiliated organizations, or those of the publisher, the editors and the reviewers. Any product that may be evaluated in this article, or claim that may be made by its manufacturer, is not guaranteed or endorsed by the publisher.

## Abbreviations

SCD: Sickle Cell Disease
HSCT: Hematopoietic Stem Cell Transplantation
HCP: Health Care Professional
SDM: Shared Decision-making
TDT: Transfusions Dependent Thalassemia.
