# Supplementary material for: How to Facilitate Decision-Making for Hematopoietic Stem Cell Transplantation in Patients With Hemoglobinopathies. The Perspectives of Healthcare Professionals
Source: Front Pediatr. 2021 Aug 18;9:690309. doi: 10.3389/fped.2021.690309 (PMC8416427; doi:10.3389/fped.2021.690309)
Supplement: Supplementary file 1 [file Table_1.docx]

## **Supplementary Table S1, Patient cases**

| **Case 1**  Timothy* is a 10 years old boy and diagnosed with SCD through neonatal screening. His brother and grandfather also have SCD; his brother is currently doing very well and has no SCD complaints. Grandfather, instead, often is admitted to the hospital with SCD complaints. Timothy himself has had years of several admissions because of severe crises; after increasing hydroxyurea is went a bit better. Recently, Timothy’s mom read on social media about the possibility of a cure for SCD, and she asked the pediatric hematologist for more information. The hematologist invited the family for a conversation about the possibility of HSCT. He explained, in general, the process of transplantation and answered many questions of the parents and Timothy. He furthermore tried to be neutral in not giving his own opinion specifically for Timothy and offered to refer them to an HSCT specialist. More specific and detailed information could help the family in considering the HSCT themselves. Another patient of this hematologist was transplanted recently and had to deal with severe HSCT-complications. A few months later, the HSCT specialist informed the family about SCD in general and the process of HSCT, including all its possible complications. This specialist underlines her responsibility to give them all trustful information and that the family had to decide for themselves. The family was impressed by the impact of an HSCT but happy to know all the possibilities. They eventually decided to proceed with the current supportive care. They keep the options open for the future depending on how the illness develops, and for an older age of Timothy, to make it possible he decides himself. |
| --- |
| **Case 2**  Twelve years old Lauren* is diagnosed with SCD as a baby. In the past years, she was admitted several times for severe crises, including three times with an acute chest syndrome. Her pediatric hematologist, with extensive experience in HSCT, referred her to an HSCT center. The hematologist joined the family in the conversation with the HSCT specialist. The family heard all information about the HSCT, including all its possible complications. Gene therapy was discussed as a possible future possibility. The family was anxious after hearing everything that could go wrong and feel the threat of the SCD in the possibility of a severe outcome of another chest syndrome. The little brothers of Lauren were not yet examined for HLA-matching. The parents preferred to protect them from medical procedures. A second conversation with more details followed, and Lauren is transplanted with a matched unrelated donor and currently doing well. |
| **Case 3**  Eight years old Boris* had transfusion-dependent thalassemia and went for blood transfusion every three weeks. The disease had a major impact on the family's wellbeing. Boris' siblings are not HLA-identical, and no matched unrelated donor is available. The parents' greatest wish is a cure for Boris. After being informed in the HSCT center, including the possibility of haplo-transplantation, the family returned after a few months for a follow-up conversation. They had considered the possibility of haplo-donation and now described their wish to go for as little as possible chemotherapy and the best possible donor. They explained their desire for another child and they had explored the possibilities. The HSCT-specialist supported the family is their choice. Two years later, Boris is transplanted with his HLA-identical sibling and is currently doing well. |
| **All patient identifiers are fictitious* |
