# Supplementary material for: How to Facilitate Decision-Making for Hematopoietic Stem Cell Transplantation in Patients With Hemoglobinopathies. The Perspectives of Healthcare Professionals
Source: Front Pediatr. 2021 Aug 18;9:690309. doi: 10.3389/fped.2021.690309 (PMC8416427; doi:10.3389/fped.2021.690309)
Supplement: Supplementary file 2 [file Table_2.docx]

Supporting Information Table S2 Consolidated criteria for reporting qualitative studies (COREQ): 32-item checklist

| ***No - Item*** | ***Guide question*** | ***Description*** |
| --- | --- | --- |
| **Domain 1: Research team and reflexivity** | | |
| Personal Characteristics | | |
| 1. Interviewer/facilitator | Which authors conducted the interview or focus group? | Page 4, data collection: first author (HM). |
| 2. Credentials | What were the researcher’s credentials? | 1^st^ author: RN - MSc  2^nd^ author: RN- MSc  3^rd^ author: MD- PhD  4^th^ author: MD- PhD  5^th^ author: MD- PhD |
| 3. Occupation | What was their occupation at the time of the study? | 1^st^ author: Pediatric hematology nurse and PhD-student  2^nd^ author: Obstetric nurse  3^rd^ author: Pediatrician endocrinologist / Ethicist / Professor  4^th^ author: Pediatrician immunologist / Professor  5^th^ author: Pediatrician / hematologist |
| 4. Gender | Was the researcher male or female? | Page 4, data collection: female. |
| 5. Experience and training | What experience or training did the researcher have? | Page 4, data collection: RN, MSc. |
| Relationship with participants | | |
| 6. Relationship established | Was a relationship established prior to study commencement? | Page 4, data collection: yes, most participants were familiar with the researcher. |
| 7. Participant knowledge of the interviewer | What did the participants know about the researcher? | Page 4, data collection: a pediatric hematology nurse working at the HSCT unit. |
| 8. Interviewer characteristics | What characteristics were reported about the interviewer/facilitator? | Page 4, data collection: HSCT nurse involved in transplantation process and experienced in interviewing. |
| **Domain 2: study design** | | |
| Theoretical framework | | |
| 9. Methodological orientation and Theory | What methodological orientation was stated to underpin the study? | Page 4, study design: qualitative interview study.  Page 4, data analysis: thematic analysis. |
| Participant selection | | |
| 10. Sampling | How were participants selected? | Page 4, population and recruitment: purposive sample. |
| 11. Method of approach | How were participants approached? | Page 4, sample: during regular meetings and by mail. |
| 12. Sample size | How many participants were in the study? | Page 5, results: Eighteen healthcare professionals. |
| 13. Non-participation | How many people refused to participate or dropped out? Reasons? | Page 5, results: none. |
| Setting | | |
| 14. Setting of data collection | Where was the data collected? | Page 4, data collection: most at the healthcare professionals’ office and one by phone. |
| 15. Presence of non-participants | Was anyone else present besides the participants and researchers? | Page 4, data collection, individual interviews. |
| 16. Description of sample | What are the important characteristics of the sample? | Page 4, results: 18 healthcare professionals: ten (paediatric) haematologists from referring centres, five (paediatric) transplantation specialists from HSCT centres, and three nurse specialist from referring centres.  Table 2, listed in characteristics table. |
| Data collection | | |
| 17. Interview guide | Were questions, prompts, guides provided by the authors? Was it pilot tested? | Table 1, interview topics.  Page 4: data collection: semi-structured interviews with open ended questions. The topic list was evaluated and adjusted during the process. |
| 18. Repeat interviews | Were repeat interviews carried out? If yes, how many? | None |
| 19. Audio/visual recording | Did the research use audio or visual recording to collect the data? | Page 4, data collection: interviews were audio recorded. |
| 20. Field notes | Were field notes made during and/or after the interview or focus group? | Page 4, data collection: observational memos were made describing the setting, atmosphere, circumstances, and the researchers’ reflections on the interview themes. |
| 21. Duration | What was the duration of the interviews or focus group? | Page 5, results: on average 38 minutes (range 27-57 min). |
| 22. Data saturation | Was data saturation discussed? | Page 4, data collection: theoretical saturation was reached after inclusion of 17 interviews, the next interview did not add to the themes and no new themes emerged. |
| 23. Transcripts returned | Were transcripts returned to participants for comment and/or correction? | No. |
| **Domain 3: analysis and findings** | | |
| Data analysis | | |
| 24. Number of data coders | How many data coders coded the data? | Page 5, data analysis: two (HM and HZ). |
| 25. Description of the coding tree | Did authors provide a description of the coding tree? | No |
| 26. Derivation of themes | Were themes identified in advance or derived from the data? | Page 5, results: derived from the data. |
| 27. Software | What software, if applicable, was used to manage the data? | Page 5, data analysis: ATLAS.ti |
| 28. Participant checking | Did participants provide feedback on the findings? | Page 9, the results were presented on a basic level during a meeting with the HCPs and shortly discussed. |
| Reporting | | |
| 29. Quotations presented | Were participant quotations presented to illustrate the themes / findings? Was each quotation identified? | Table 3, illustrative quotes. |
| 30. Data and findings consistent | Was there consistency between the data presented and the findings? | Page 5-8, results.  Table 3. |
| 31. Clarity of major themes | Were major themes clearly presented in the findings? | Page 5-8, results.  Table 3. |
| 32. Clarity of minor themes | Is there a description of diverse cases or discussion of minor themes? | Page 5-8, results.  Table 3. |
